# Prevalence of lower urinary tract infection in South Indian type 2 diabetic subjects

**DOI:** 10.4103/0971-4065.65315

**Published:** 2010-04

**Authors:** J. Janifer, S. Geethalakshmi, K. Satyavani, V. Viswanathan

**Affiliations:** M.V. Hospital for Diabetes and Diabetes Research Centre (WHO Collaborating Centre for Research Education and Training in Diabetes), No. 4, Main Road, Royapuram, Chennai - 600013, India

Sir,

We thank Dr. Burman for his comments on our recent paper. We agree with most of the points made by him.

Of the fungal urinary tract infections (UTIs), candidal vulvovaginitis is more common in diabetic patients. Screening for Candida colonies in the routine culture and sensitivity will help in identifying all Candida species in urine specimens.

So far as obtaining specimens for urinalysis is concerned, the suprapubic aspirate is ideal as it avoids urethral contamination, but is an invasive procedure. It is usually reserved for infants and for some patients from whom it is difficult to obtain urine specimens.[1]

Increasing the duration of antibiotic treatment to 7–14 days may be necessary to treat the UTI. However, this needs to be confirmed by long-term prospective studies.

Finally, circumcision is beneficial in male patients, but personal hygiene is very important in female patients as their vagina is located close to the anus and can be colonized by members of the fecal flora.
